# Comparison of pesticide residues in surface water and ground water of agriculture intensive areas

**DOI:** 10.1186/2052-336X-12-11

**Published:** 2014-01-07

**Authors:** Summaiya Z Lari, Noor A Khan, Kavita N Gandhi, Tejal S Meshram, Neeta P Thacker

**Affiliations:** 1 CSIR-National Environment and Engineering Research Institute, Nehru Marg, Nagpur, India

**Keywords:** Pesticide residue, Ground water, Surface water, Organochlorine, Organophosphate

## Abstract

The organochlorines (OClPs) and organophosphates (OPPs) pesticides in surface and ground water having intensive agriculture activity were investigated to evaluate their potential pollution and risks on human health. As per USEPA 8081 B method, liquid-liquid extraction followed by Gas-Chromatographic technique with electron capture detector and mass selective detector (GC-MS) were used for monitoring of pesticides. Among organochlorines, α,β,γ,δ HCH’s, aldrin, dicofol, DDT and its derivatives, α,β endosulphan’s and endosulphan-sulphate were analysed; dichlorovos, ethion, parathion-methyl, phorate, chlorpyrifos and profenofos were determined among organophosphates.

As compared to ground water, higher concentrations of OClPs and OPPs were found in surface water. Throughout the monitoring study, α - HCH (0.39 μg/L in Amravati region),α - endosulphan (0.78 μg/L in Yavatmal region), chlorpyrifos (0.25 μg/L in Bhandara region) and parathion-methyl (0.09 μg/L in Amravati region) are frequently found pesticide in ground water, whereas α,β,γ-HCH (0.39 μg/L in Amravati region), α,β - endosulphan (0.42 μg/L in Amravati region), dichlorovos (0.25 μg/L in Yavatmal region), parathion-methyl (0.42 μg/L in Bhandara region), phorate (0.33 μg/L in Yavatmal region) were found in surface water.

Surface water was found to be more contaminated than ground water with more number of and more concentrated pesticides. Among pesticides water samples are found to be more contaminated by organophosphate than organochlorine. Pesticides in the surface water samples from Bhandara and Yavatmal region exceeded the EU (European Union) limit of 1.0 μg/L (sum of pesticide levels in surface water) but were within the WHO guidelines for individual pesticides.

## Introduction

If the credits of pesticides include enhanced economic potential in terms of increased production of food and fiber, and amelioration of vector-borne diseases, then their debits have resulted in serious health implications to man and his environment. There is now overwhelming evidence that some of these chemicals do pose potential risk to humans and other life forms and unwanted side effects to the environment [1,2]. Ideally a pesticide must be lethal to the targeted pests, but not to non-target species, including man. Unfortunately, this is not so. The controversy of use and abuse of pesticides has surfaced. The rampant use of these chemicals, under the adage, “if little is good, a lot more will be better” has played havoc with human and other life forms. In India, the first report of poisoning due to pesticides was from Kerala in 1958, where over 100 people died after consuming wheat flour contaminated with parathion [3].

India ranks 10th in the world in pesticide consumption, as its total consumption amounts to about 500 million tonnes. India is presently the largest manufacturer of basic pesticides among the South Asian and African countries, with the exception of Japan. The Indian pesticides market is the 12th largest in the world with a value of US$0.6 bn, which is 1.6% of the global market pie [4]. During one survey in India, 58% of drinking water samples drawn from various hand pumps and wells around Bhopal were contaminated with organochlorine pesticides above the EPA standards [5]. The majorities of the organochlorine pesticides are very persistent in environmental media and generate severe adverse health impacts.

Vidarbha region in Maharashtra comprises 11 districts. Livelihood of around 65% rural population of this region is dependent on agriculture and allied activities [6]. Groundwater from dug well, handpump, tube wells and water from rivers like Wainganga, Wardha, Purna and Kanhan which are all tributaries of Godavari River, are the major source of drinking water for Vidarbha region.

Drinking water from many parts of India has been reported with presence of organochlorine and organophosphate pesticide residues [7-9]. Limited information has been reported on pesticide contamination of Vidarbha region. All these considerations suggested the need to implement the current study.

The study targets the assessment of pesticide residues in water bodies of agriculture intensive areas and comparison of pesticide residues in groundwater and surface water. These measurements can be used as baseline levels to monitor the future changes and to predict their future impact on the population of the area.

Besides these monitored pesticides, others can be present in Vidarbha surface and ground water. Further monitoring is hence needed and a seasonal trend to be established as the study is restricted to particular seasons only.

## Materials and methods

### Study area

**Figure 1:**
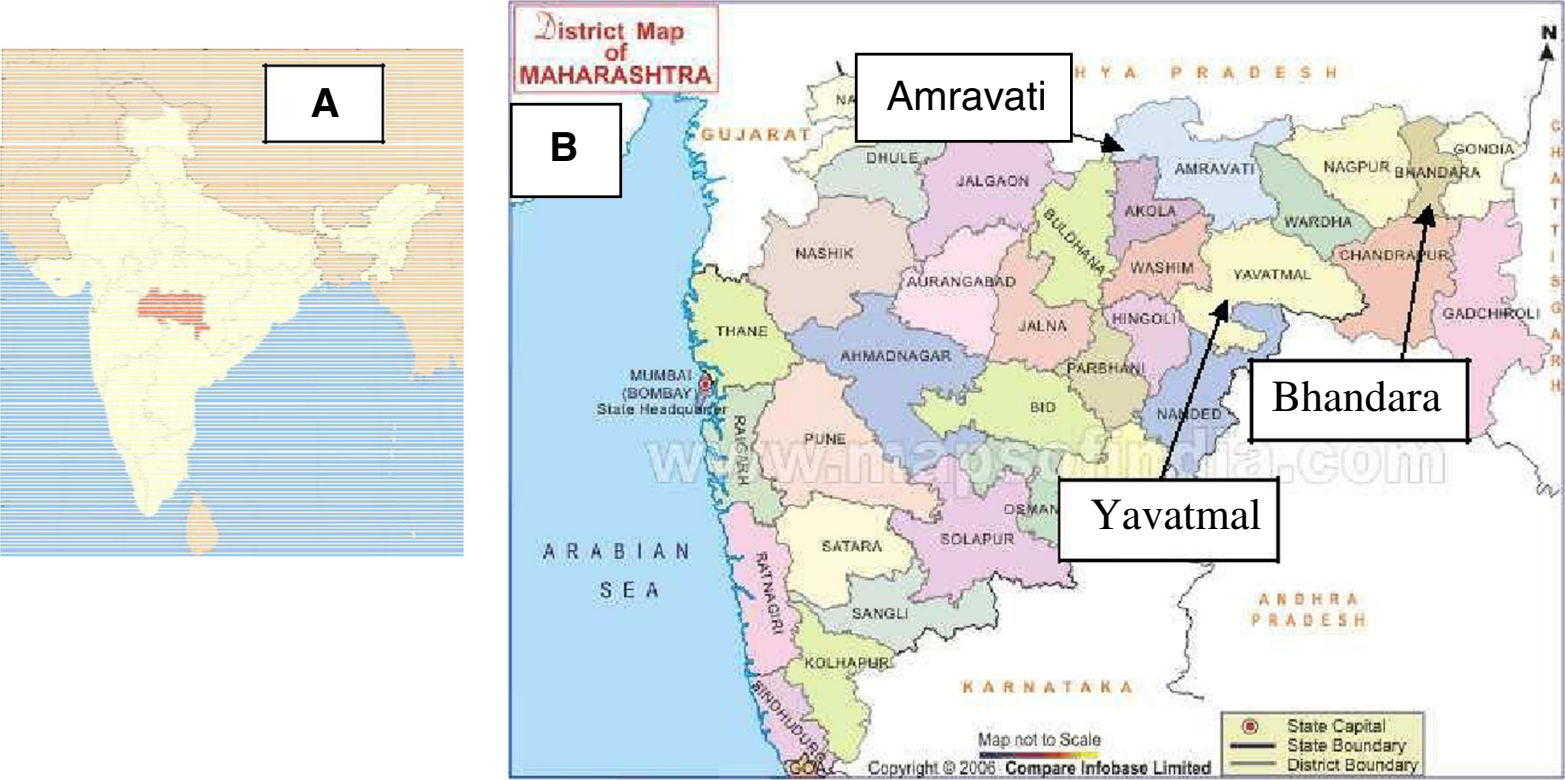
**Study area and sampling locations. (A)**: Map of India highlighting Vidarbha region, **(B)**: Sampling location of Amravati, Yavatmal and Bhandara.

Figure 1 shows study area and sampling locations. Vidarbha is the eastern region of Maharashtra state, India (Figure 1A). It occupies 31.6% of total area and holds 21.3% of total population of Maharashtra [10]. It is one of the major agriculture intensive areas of Maharashtra. The major cultivated crop species of Vidarbha region are gram, cotton, chilli, brinjal, tur, tomato, wheat, lemon, orange, soyabean, jowar, bajra and maize. As a result, some pesticides are extensively used in these crops like endosulphan, chlorpyrifos, dicofol, dichlorovos, phorate, malathion, triazophos, etc. Surface and ground water areas of Vidarbha region of Maharashtra state – Bhandara (3717 sq km), Amravati (12212 sq km) and Yavatmal (13584 sq km) (Figure 1B). For ground water study the samples of tube well, open well and hand pumps from agricultural sites had been collected seasonally from 10 different sampling sites. River and lake water had been collected from 6 different sampling sites. Field samples were analyzed for organochlorine (OClP) and organophosphate pesticides (OPPs), with each set of samples, one blank and one control sample.

### Water sampling, collection and storage

Collection of water samples were performed out from September 2011 to July 2012. Total numbers of 156 water samples were collected from different sites. Grab sampling was done. Samples were collected in 1 liter amber colored glass bottles. Sampling bottles were rinsed well with water and were carefully filled to overflowing, without trapping air bubbles in sealed bottles. The samples were transported in cool-box with ice packs. Preparation of the containers included washing with detergent, rinsing with tap water, ultrapure water (Millipore) and air-dried. After transportation to the laboratory, samples were stored at 4°C and extraction was mostly done within 48 h.

### Analytical methodology

#### Chemicals and reagents

All pesticide analytical standards were procured from Dr. Ehrenstorfer Gmbh, Germany. The solvents used for the extraction were obtained from Merck (HPLC grade for Chromatography). Individual pesticide stock standard solutions were prepared by exact weighing of high-purity substances in 10 mL volumetric flasks and filled up with an appropriate solvent like acetone and n-hexane. All stock standard solutions were stored in a deep freezer protected from light at -20°C. An intermediate and working standard of suitable concentration was made from the stock as and when required.

#### Sample extractions and analysis

Water samples were filtered with Whatman no. 1 filter paper to remove debris. 800 mL of water sample was transferred into a 1 liter glass-separating funnel. Then, 80 g of NaCl was added to produce a salt out effect. It was thoroughly mixed by inverting the flask three to four times. The sample was extracted thrice with 160 mL dichloromethane (80:40:40); shaken for 3–4 min each time with periodic venting. The combined organic phase was dried by passing it through anhydrous Na_2_SO_4_. The organic phase was concentrated to 3–5 mL in a vacuum rotary evaporator (Heidolph) and further dried under a gentle stream of nitrogen in a Turbovap (Caliper Science) low volume concentrator. The sample was reconstituted in 1 mL of n-hexane and 1 μL of the aliquot was analyzed by GC-ECD (Gas Chromatography-Electron Capture detector) and GC–MS (Gas Chromatography-Mass Spectroscopy).

#### GC and GC-MS Analysis

The pesticide residues were analyzed by gas chromatograph equipped with ^63^Ni electron capture detector (SHIMADZU GC-2010) and presence of pesticides was confirmed by Varian Saturn 2200 GC-MS.

The determinations of pesticides residue had been performed following U.S. Environmental Protection Agency (USEPA), Method- 8081 B and self-modified laboratory method using GC-SHIMADZU with Electron Capture Detector. The column specifications and operating conditions are given in Table 1.

**Table 1:** Gas Chromatograph conditions for pesticide analysis

**Item**	**Condition**	
GC	Make – SHIMADZU	
	Model –GC-2010 (Auto sampler)	
Detector	Electron Capture Detector (ECD)	
Column	DB-5 column of 30 m length, 0.25 mm inner diameter (ID) &	
	film thickness of 0.25 μm	
	Organochlorines	Organophosphates
Injection volume	1 μL	1 μL
Injector temperature	250°C	220°C
Detector temperature	300°C	270°C
Carrier gas flow rate	0.95 mL/min	0.87 mL/min
Oven programming	70°C (2 min hold) to 160°C	150°C (1 min hold) to 225°C
	@ 15°C/min to 270°C @ 5°C/min (18 min hold)	@ 5°C/min (10 min hold)
Total run-time	48 min	26 min

A Varian Saturn 2200 gas chromatograph mass spectrometer was used for confirmation of pesticide analysis. The injection port temperature was set at 250°C and a liner with a plug of glass wool was installed. An amount of 1 μL of the concentrated extracts was injected in split mode (1:5). Helium was used as the carrier gas at a flow rate of 0.94 mL/min. The pesticides were separated with a 50.10 min oven temperature program built as follows: initial temperature 40°C (hold 2 min), increase at 25^◦^C min^-1^ to 130°C (hold 0 min), increase at 12^◦^C min^-1^ to 180°C (hold 0 min) and finally increase at 3^◦^C min^-1^ to 280°C (hold 7 min). The mass spectrometer was operated in the electron impact (70 eV) selected ion monitoring (SIM) mode. The temperature of the injector and interface were 200°C and 250°C, respectively.

### QA/QC

**Figure 2:**
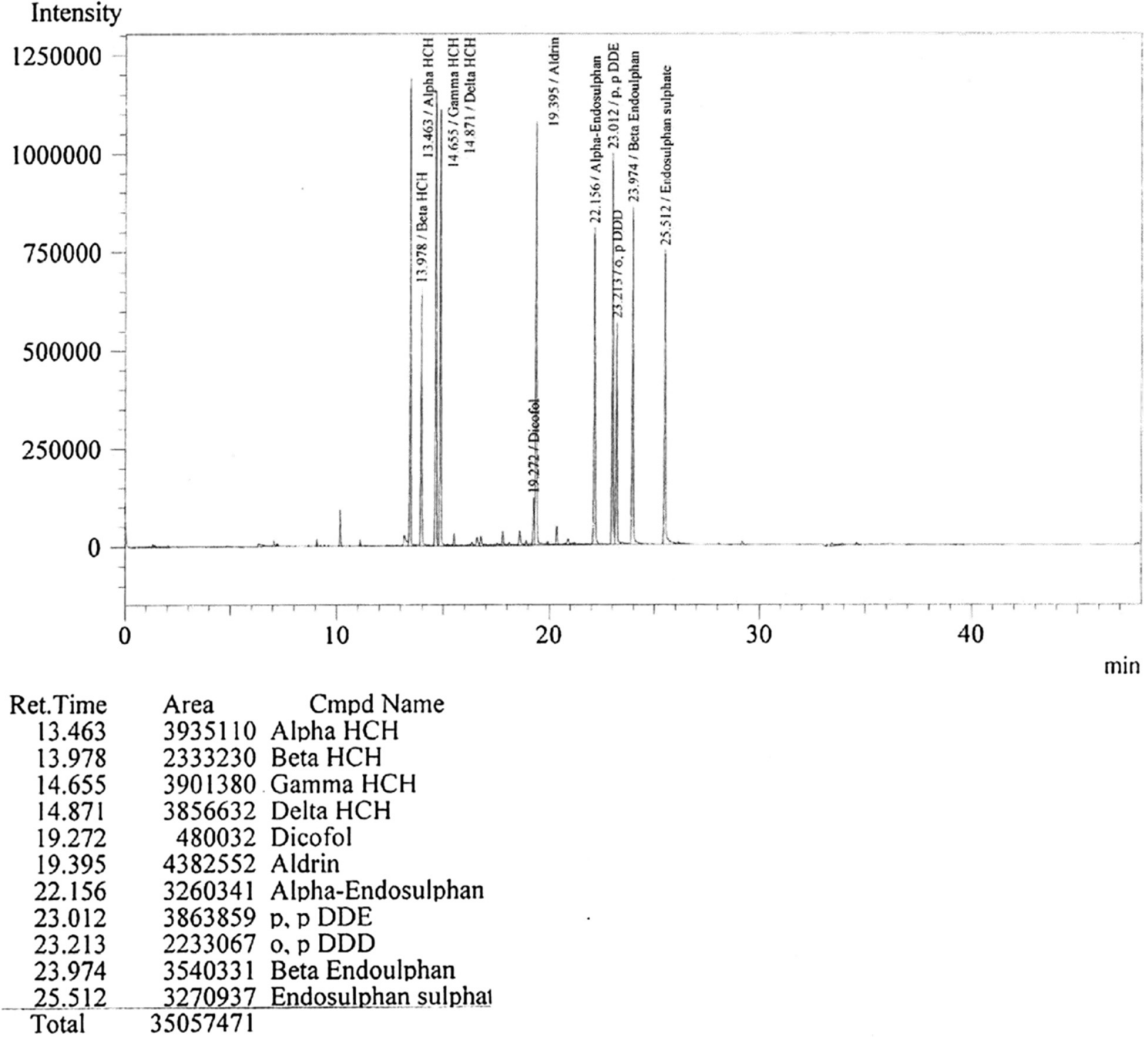
Chromatogram showing retention time for Organochlorine.

The chromatogram showing retention times for organochlorines shown in Figure 2. Recovery studies were performed by adding known amount of standard mixture of pesticides in ultra pure water (Millipore, Milli DI). The average percent-recovery of organochlorine pesticides was estimated at five concentration levels (0.25, 0.5, 1, 2.5 and 5 ppb). Validation studies for each concentration were done in triplicates. The average recovery (%), range of relative standard deviation (RSD) of pesticides at five concentration levels is given in Table 2. The limit of detection (LOD) and limit of quantitation (LOQ) have also been determined. Detection limits for organochlorine pesticides varied between No GuidelineValue (well below health concern in drinking water) to 30 μg/L [11].

**Table 2:** QA/QC data of organochlorine pesticides

**Organochlorine pesticides**	**LOD (μg/L)**	**LOQ (μg/L)**	**Average % recovery**	**Range of RSD**
Alpha-HCH	0.067	0.226	70.51	0.011 - 0.041
Beta-HCH	0.045	0.15	74.91	0.009 - 0.017
Gamma-HCH	0.058	0.196	71.12	0.005 - 0.039
Delta-HCH	0.064	0.213	82.42	0.047 - 0.127
Dicofol	0.039	0.13	96.24	0.014 - 0.053
Aldrin	0.047	0.157	69.72	0.006 - 0.12
Alpha-endosulphan	0.042	0.14	83.99	0.009 - 0.022
pp’ DDE	0.041	0.135	74.5	0.005 - 0.018
op’ DDD	0.046	0.153	72.08	0.012 - 0.091
Beta-endosulphan	0.046	0.155	89.49	0.013 - 0.048
Endosulphan sulphate	0.07	0.235	100.93	0.014 - 0.146

## Results & discussions

The result of analysis of water samples from Vidarbha region (Table 3) affirmed the presence of HCH isomers (alpha, beta, gamma, delta-hexachlorocyclohexane), Endosulphan, DDT (*p*, *p*’-dichlorodiphenyltrichloroethane), Dichlorovos, Chlorpyrifos, Phorate, etc.

**Table 3:** Minimum to maximum range of concentrations of pesticides (μg/L)

**Samplingsites/pesticides**	**Bhandara**		**Amravati**		**Yavatmal**	
	**Ground water**	**Surface water**	**Ground water**	**Surface water**	**Ground water**	**Surface water**
HCH	ND-0.06	ND-0.06	ND-0.39	ND-0.39	ND-0.08	ND
Endosulphan	ND-0.72	ND-0.08	ND-0.60	ND-0.42	ND-0.78	ND
DDT	ND	ND	ND	ND	ND	ND
Dichlorovos	ND-0.09	ND-0.20	ND-0.08	ND-0.20	ND-0.07	ND-0.25
Chlorpyrifos	ND-0.25	ND-0.44	ND-0.11	ND-0.26	ND-0.18	ND-0.44
Phorate	ND	ND-0.31	ND	ND-0.19	ND	ND-0.33
Parathion-methyl	ND-0.03	ND-0.42	ND-0.09	ND-0.15	ND-0.02	ND-0.17

**Figure 3:**
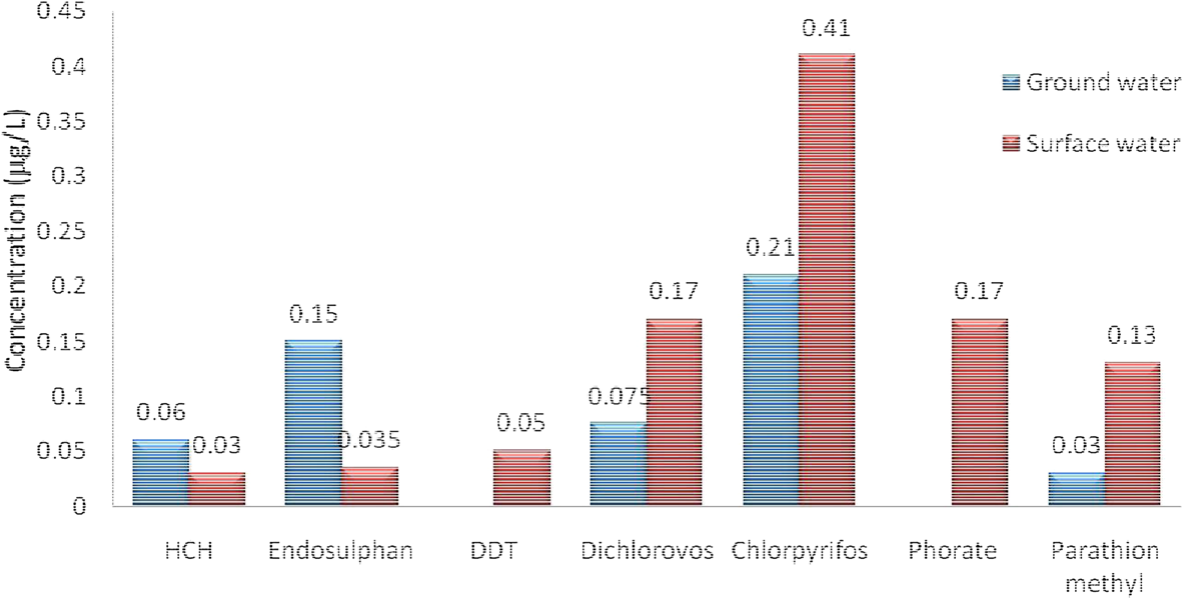
Average of individual pesticide detected in Bhandara region.

**Figure 4:**
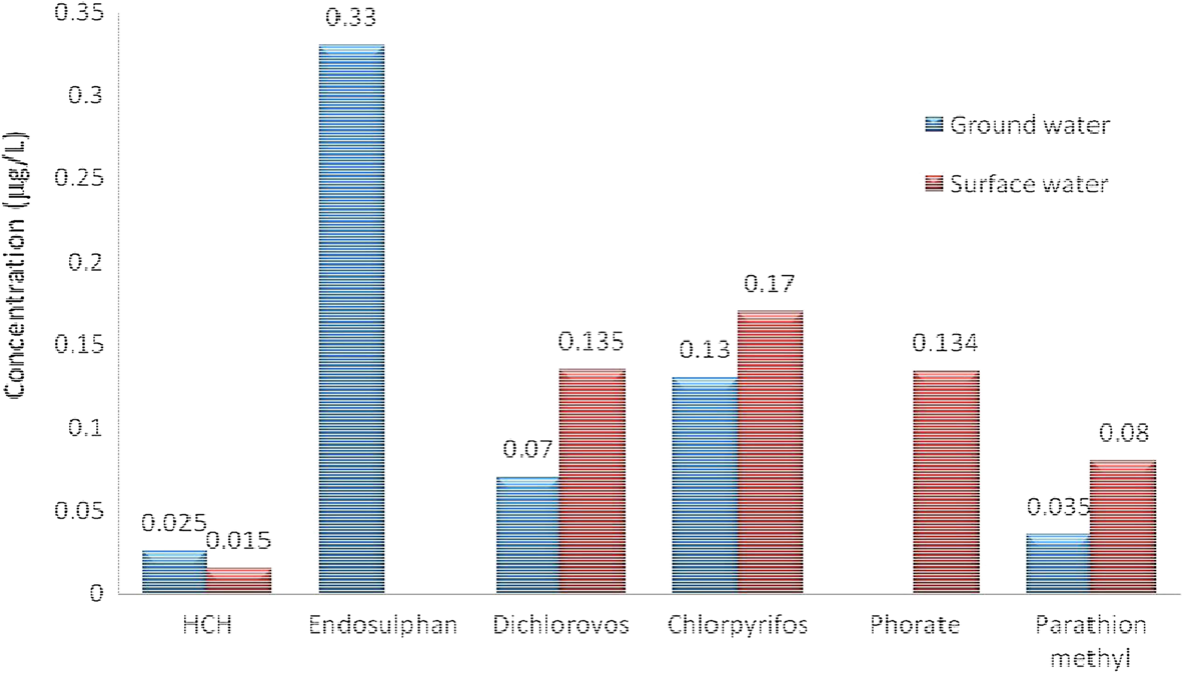
Average of individual pesticide detected in Yavatmal region.

Surface water was found to be more contaminated than ground water with more number of and more concentrated pesticide residues. Among the various pesticides analyzed Dichlorovos, Chlorpyrifos, Phorate and Parathion-methyl were consistently found pesticides. The highest concentration of 0.44 μg/L was observed for Chlorpyrifos in Bhandara and Yavatmal region and 0.42 μg/L for Parathion-methyl in Bhandara region (Table 3). Pesticides in the surface water samples from Bhandara and Yavatmal region (Figures 3, 4) exceeded the EU (European Union) limit of 1.0 μg/L (sum of pesticide levels in surface water). In accordance with European Economic Commission for drinking water, the total pesticide level should not exceed 0.5 μg/L and individual pesticide not greater than 0.1 μg/L [12] but were within the guideline value of individual pesticides assigned by WHO [11].

An Indo-Dutch study have shown alarming levels of pesticides in the Yamuna water supplies to Delhi. OClPs like aldrin, BHC, DDT, dieldrin were detected in the range of 0.001 - 1.064 μg/L [9]. BHC and DDT residues were also detected in the waters of Keoladeo National park and Bharatpur, Rajasthan in the range of 0.58 and 3.86 μg/L [13]. A study reports the concentration levels and distribution pattern of the organochlorine pesticide (OCPs) residues in the soil and surface water samples collected from the northern Indo-Gangetic alluvial plains. The results showed contamination of soil and surface water of the region with several persistent organic pesticides. The total OCPs level ranged from 0.36–104.50 ng/g and 2.63–3.72 μgL in soil and surface water samples, respectively [14].

The ground water samples analyzed from agricultural area were mostly contaminated with atleast one of the isomer of HCH and Endosulphan among OClPs. A highest of 0.39 μg/L HCH been reported in Amravati district, predominant being gamma-HCH. The pesticide pollution by Endosulphan (sum of all isomers) was found to be maximum in ground water sample analysis with maximum value as 0.72 μg/L, 0.6 μg/L and 0.78 μg/L in Bhandara, Amravati and Yavatmal region respectively. Among organophosphates Diclorovos and Chlorpyrifos accounted with maximum value reported for Chlorpyrifos as 0.25 μg/L at Bhandara region.

India is one of the few remaining countries still engaged in the large scale manufacture, use and export of some of the toxic chlorinated pesticides, such as *p*, *p*’-dichlorodiphenyltrichloroethane (DDT), hexachlorocyclohexane (HCH) and pentachlorophenol (PCP). The cumulative consumption of the pesticide, hexachlorocyclohexane (HCHs), in India until 1985 was 575,000 tons and since then about 45,000 tons of HCHs has been used annually. The usage of DDT and HCH continued till recently [15]. After almost forty years of extensive use worldwide, there has been a gradual replacement of technical hexachlorocyclohexane (HCH) by lindane (gamma-HCH). No significant uses of technical HCH have been reported after 2000. However releases into the environment may also occur from lindane production as well as from hazardous waste sites, landfills and contaminated sites [16]. Organophosphorous compounds have overtaken organochlorine compounds as the most used insecticides in the recent decade [15], in described study also the overall contamination in water samples by organophosphorous had overtaken organochlorine.

A number of researchers have reported pesticides and heavy metals in drinking and groundwater in different parts of India. The main source of drinking water in rural areas that is ground water in Ambala and Gurgaon district and surface water supply in Hisar district of Haryana was found to be contaminated by isomers of HCH, Endosulphan and metabolites of DDT [7]. There are a few reports on the presence of OPPs residues in different rivers of India [8]. Water samples collected from 28 domestic well supplies of the Hyderabad city shows contamination levels of organochlorine pesticides. DDT was found to range between 0.15 and 0.19 μg/L, β-Endosulfan ranges between 0.21 and 0.87 μg/L, α-Endosulfan ranges between 1.34 and 2.14 μg/L and Lindane ranges between 0.68 and 1.38 μg/L respectively [17].

The most frequently detected pesticides in treated water plants of Delhi region are lindane, p,p’-DDE and endosulphan I and endosulphan II. These pesticides are found in 73% of the 85 samples analyzed during 2000–2005 [18]. The levels of OClPs , lindane, p,p’ DDT, p,p’ DDE, p,p’ DDD, endosulphan I, endosulphan II monitored and reported in urban water resources, viz; river, lake, tubewells and intake and final water treatment plants of major cities in India like, Delhi, Mumbai and Nagpur [19].

The levels of residues found are in agreement with the reviewed data presented by other researchers, concerning studies of ground and surface water exposure to pesticides in different regions of India and with the data collected from local farmers from respective regions, crop pattern and pesticide usage. To the best of knowledge no report has been submitted till date reporting the pesticide residue analysis of Vidarbha region. Hence, stated study provides the analytical methodology and data of pesticide analysis of selected region. An extension of study areas and number of pesticides analysed, would be considered in future work. The implementation of multi-residue methods and also of automated techniques like SPME-GC-MS to improve analytical conditions would also be targeted.

## Conclusions

Studies on pesticide pollution in the above referred work bespeak the presence of pesticide residues in water bodies of agriculture intensive areas of Vidarbha region.

**Figure 5:**
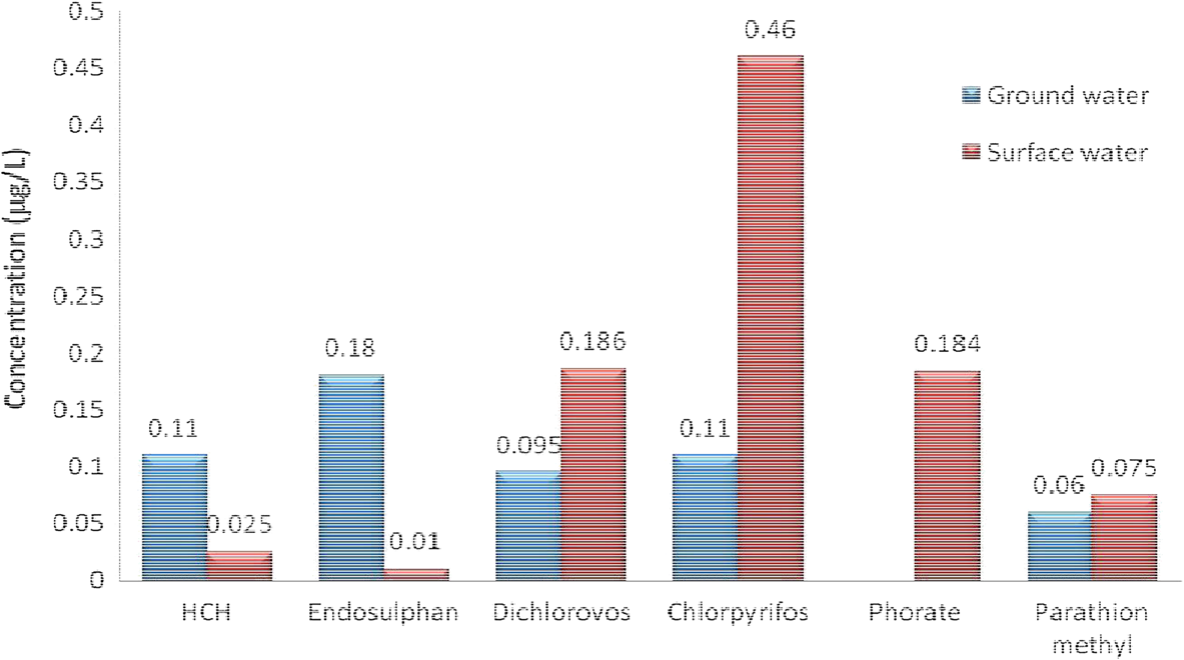
Average of individual pesticide detected in Amravati region.

• Surface water was found to be more contaminated than ground water. The values of average of individual OPPs levels show highest concentration of 0.41 μg/L and 0.46 μg/L for Chlorpyrifos in Bhandara and Amravati region respectively (Figures 3, 5).

• The ground water samples were mostly contaminated with atleast one of the isomer of HCH, endosulphan, diclorovos and chlorpyrifos. The pesticide pollution by sum of all isomers of Endosulphan was found to be maximum in ground water sample analysis with maximum value as 0.33 μg/L in Yavatmal region (Figure 4) and Chlorpyrifos as 0.21 μg/L at Bhandara region (Figure 3).

## Abbreviations

(OClPs): Organochlorines; (OPPs): Organophosphates; (USEPA): U.S. Environmental Protection Agency; (GC-ECD): Gas-Chromatographic technique with electron capture detector; (GC-MS): Gas-Chromatographic technique with mass selective detector; (α, β, γ, δ-HCH): Alpha, beta, gamma, delta-hexachlorocyclohexane; (DDT): *p*, *p*’-dichlorodiphenyltrichloroethane; (EU): European Union; (WHO): World Health Organization; (SIM): Selected ion monitoring; (RSD): Relative standard deviation; (LOD): Limit of detection; (LOQ): Limit of quantitation.

## Competing interests

The authors declare that they have no competing interests.

## Authors’ contributions

The overall implementation of this study including the design, sample collection and preparations, laboratory experiments, data analysis, and manuscript preparation was performed by all the authors. All the authors have made extensive contribution into the review and finalization of this manuscript. All authors have read and approved the final manuscript.
